# VITAL INVOLVEMENT: A SOURCE OF REALISTIC OPTIMISM FOR OLDER ADULTHOOD

**DOI:** 10.1093/geroni/igz038.1516

**Published:** 2019-11-08

**Authors:** Helen Q Kivnick

**Affiliations:** University of Minnesota School of Social Work, Saint Paul, Minnesota, United States

## Abstract

Vital Involvement (VI) was initially proposed (Erikson et al., 1986) as one of three principles around which lifelong healthy psychosocial development takes place. As more recently elaborated, VI has come to describe a person’s meaningful, reciprocal engagement with the world outside the integrating “self.” It is through VI that the person engages in healthy psychosocial development throughout life, including balancing Older Adulthood’s focal tension between Integrity and Despair. This life stage is widely associated with the physical, cognitive, and social losses, and societal constraints that give rise to later-life despair. However, VI functions as a lifelong psychosocial model for the meaningful environmental engagement that supports later life’s wisdom and integrity. Notably few films present an integrated view of older adulthood’s losses along with opportunities. But those few can be a source of optimism to elders for whom VI may not be intuitive, but who can learn its practice.

